# Management of Recurrent Urethral Strictures: A Therapeutic Challenge

**DOI:** 10.7759/cureus.12253

**Published:** 2020-12-24

**Authors:** Mashood Iqbal, Uzzam Ahmed Khawaja

**Affiliations:** 1 Internal Medicine, Jinnah Medical College Hospital, Karachi, PAK; 2 Internal Medicine, Jinnah Medical and Dental College, Karachi, PAK; 3 Clinical and Translational Research, Larkin Community Hospital, South Miami, USA

**Keywords:** recurrent urethral strictures, urethral stenting, urethrotomy, urethroplasty

## Abstract

Urethral stricture disease is relatively common, and its management remains a therapeutic challenge for urologists despite recent advancements in endoscopic and reconstructive surgery. The majority of the strictures are acquired from injury or infection. Urethral stent implantation, a minimally invasive procedure, can be safely and effectively used as a primary surgical procedure in treating recurrent urethral stricture. Herein, we present a case of a 43-year-old male patient with complaints of intermittent urination, oliguria, and incomplete voiding with urinary frequency. Further urological investigations, a uroflowmetry, and a urethrogram were carried out. Oliguria, along with a mid-bulbar urethral stricture at the previous excision anastomotic site, was diagnosed. Despite a higher success rate of urethroplasty and temporary stent placement, urethral stricture recurrences are still an occurring entity. No definite therapeutic strategy has been adopted to evaluate and approach the morbidity effectively. Implementation of an effective primary procedure with minimally based complications should be generated to avoid future stricture recurrences. Larger-scale studies involving urethral stricture patients can gather sufficient data to obtain a complete curative treatment option for the future.

## Introduction

Urethral stricture disease is frequently encountered clinically and accounts for substantial morbidity and cost to the medical system [1]. Frequent recurrence after initial treatment is common [2]. Urethral stent implantation has been categorized as a minimally invasive technique that can be safely and effectively used as a primary surgical procedure to treat recurrent urethral stricture [3]. According to the American Journal of Roentgenology, temporary stent placement has been considered the treatment of choice in cases of recurrent urethral strictures. High recurrences of urethral strictures with conventional treatments, including endoscopic urethrotomy and dilation, have been positively correlated. Ideal management of recurrent strictures should entail a noninvasive intervention that would ultimately result in a long-term elimination of the obstruction with minimal complications [4].

## Case presentation

A 43-year-old male patient with NKCM presented to our medicine out-patient department (OPD) with complaints of intermittent urination, decreased flow of urine, and incomplete voiding with sudden, frequent urges to urinate. The onset of symptoms was gradual, progressively worsening in nature. The patient also complained of hypogastric pain during urination and a sensation of incomplete bladder emptying after urination. However, there were no burning micturition complaints, urinary or stress incontinence, post-void dribbling, discharge, or hematuria. On examination, his vitals were stable with an unremarkable systematic examination. The patient's previous medical history revealed similar symptoms for which he had undergone optical urethrotomy twice in 2008 and 2010 and an excision anastomotic urethroplasty in 2012. 

On his recent visit, the symptoms were reanalyzed, and it was found to be a recurrent urethral stricture. Further urological investigations were carried out, including a uroflowmetry and a urethrogram. A decreased flow rate and a mid-bulbar urethral stricture at the previous excision anastomotic site were diagnosed from the investigations, respectively. The preoperative diagnosis was termed as a recurrent bulbar urethral stricture.

Optical urethrotomy was eventually decided and executed. The patient was taken to the operating room and underwent an uneventful induction of anesthesia along with an appropriate central and arterial access. Aseptic measures were successfully secured and the patient rendered in a lithotomy position. A careful cystourethroscopy, subsequently followed by the introduction of an optical urethrotome, was performed. Full-length incision of the stricture was implemented at 12'-o-clock position. Further, a 16F Foley's catheter was placed, and the patient successfully moved to the recovery room in a stable condition with no complications encountered.

## Discussion

Urethral stricture has been defined as a reduction of the urethral lumen from a scarring process resulting from trauma, localized inflammation, and iatrogenic or idiopathic pathologies. The pathology has been graded as the most common cause of obstructed urination in young adults [2]. From a statistical point of view, the disease is relatively common in men with a rate as high as 0.6% in some populations. A study conducted at the Sindh Institute of Urology and Transplantation (SIUT) in Karachi, Pakistan, reveals that the stricture morbidity comprises 3%-4% of all urologic diseases alongside 5,760 patients who visit them annually for dilatation, other treatment options, and follow-up uroflowmetry. They further claimed indoor admissions of two hundred and seventy-four urethral stricture patients in 2006 in their Institute [5]. Urethral injury and infections have been termed as the leading etiologies of urethral strictures, utterly absent in the patient we encountered. Literature also suggests iatrogenic etiology accounting for more than 45% of stricture cases, especially in patients older than 45 years old. Iatrogenic causes include transurethral resection (TUR), cystoscopy, brachytherapy, hypospadias surgery, and urethral catheterization [6].

Various management modalities of urethral stricture disease, such as internal urethrotomy, intermittent urethral dilatations, and open urethroplasties, have been well described in the literature [2,3,7]. However, clear curative roles of these interventions are still underway with unimpressive rates of success. The long-term success rate after the first internal urethrotomy is only 20%-45%. The low success rate also justifies the re-encounter of the stricture ahead in time of our patient. However, after performing a urethroplasty with a wide variety of techniques, the success rate is up to 90%-95%. Despite a high percentage of justifying urethroplasty as a gold standard procedure, its success rate dramatically decreases down to 40% in complex urethral stricture cases [2,3]. Complex stricture could have possibly been the cause of recurrence despite an excision anastomotic urethroplasty in our patient. Studies have also clearly highlighted internal urethrotomy and urethral dilatation as a causative factor for an elevated rate of recurrent urethral strictures with a low long-term efficacy [2,7].

According to the Brazilian Journal of Urology, stricture recurrence, although not related to mortality, has been described as one of the biggest urological problems encountered clinically [3]. The most common complication of a standard urethroplasty or urethral reconstruction has also evolved to be stricture recurrence, consistent with the patient we encountered [2]. Literature effectively claims how the frequent recurrence of urethral stenosis could negatively impact the patient, eventually affecting the quality of life with management still being a therapeutic challenge [3,4]. These facts highlight recurrence as a reality after primary stricture intervention and, therefore, a proper therapeutic task to deal with such patients. The latter statement is further reinforced by a study that evaluated long-term internal urethrotomy results for anterior urethral strictures. Their results demonstrated an overall recurrence rate of 68% after a single urethrotomy intervention alongside claiming an unimproved success rate after repeated urethrotomy. The study ultimately concluded the need for alternative treatments, especially after the failure of initial urethrotomy [8].

According to the literature, intermittent self-catheterization (ISC) is routinely prescribed by most urologists for the prevention of urethral stricture recurrence [2]. Another three-year randomized controlled study conducted at the Institute of Kidney Diseases, Hayatabad Medical Complex; Peshawar, Pakistan, concluded clean intermittent self-catheterization is a simple and effective choice way of reducing stricture recurrence, particularly after internal optical urethrotomy. They claimed it to be highly cost-effective and strongly linked it with less morbidity [9]. Post-operatively, the procedure has also been suggested to reduce the stricture recurrence rate [2].

The application of urethral stent, another curative intervention for stricture recurrence, has gained considerable popularity in the literature. The American Journal of Roentgenology suggests stent placement as an alternative treatment for patients with recurrent urethral strictures, whose condition is unsuited to invasive procedures [4]. Urethral stenting has been classified under permanent and temporary interventions. A negative perception of permanent stenting is its permanent placement in the urethra. Temporary urethral stenting, however, following urethral dilation or internal urethrotomy has been found to maintain urethral patency and decrease stricture recurrence, clearly without the need for permanent implantation [6]. The literature review also suggests the use of temporary urethral stent placement for recurrent bulbar urethral strictures after direct visual internal urethrotomy or dilatation as an option before deciding on urethroplasty [7]. This comparatively enables urologists to approach the patient with a minimally invasive procedure and overall compliance with the patient's satisfaction rather than surgically reconstructing the urethra.

Temeltas et al. analyze temporary urethral stent implementation, particularly in patients who have undergone numerous internal urethrotomy or dilatations [7]. This initiates a strategic plan for patients with failures from repeated urethrotomy or dilatations. He further explains that a temporary stent may eventually stabilize the stricture site during epithelization of the scarred wound from previous explorations, thereby reducing the need for further endoscopic or urethroplasty procedures [7]. Short-term stenting has also been preferred because of fewer complications encountered clinically, including migration, discomfort, incontinence, and infection. Despite an overall positive response of temporary stenting reported in the literature, a study also claimed the use of stenting as a primary curative procedure with a high rate of stricture recurrence of up to 45% in the follow-up period [10].

Contrary to what has been published, studies also strongly claim a higher success rate of permanent stent implementation than direct visual internal urethrotomy in recurrent urethral strictures; however, the complication rate after the stent placement remains very high in the literature. Another study revealed certain complications they encountered in patients after stent placement including partial stent migration: 4.3%, hyperplastic reaction: 14.9%, discomfort in implantation area: 42.6%, post-micturition dribbling: 68.1%, pain during erection: 6.4% [7].

A temporary bulbar urethral stent named Allium has been acclaimed in the literature as feasible and safe for the treatment of recurrent urethral stricture. Long-term use has led to an acceptable failure rate of 25% by an analysis. Another study also utilized Allium urethral stent on 54 patients with recurrent benign bulbar urethral strictures. However, they removed the stents three or 18 months after stent insertion with a reported success rate of 81.4% [7,10]. This allows the patients to analyze the facts on record and divert more towards the option of temporary stent placement.

Long-term results of another urethral stent, urolume, for recurrent bulbomembranous urethral strictures were analyzed in a study. The analysis concluded promising early results with applying the latter stent alongside a low late failure rate. It was also claimed as a successful treatment option for many recurrent bulbomembranous urethral strictures [11].

The use of Holmium laser urethrotomy for urethral strictures has also been mentioned in the literature with a success rate of 78%. Sixty-nine patients underwent the latter intervention, of which 54 had no recurrence on six-month follow-up [5]. Another well-established modality has also gained considerable weightage in the literature. The use of Buccal Mucosal Graft (BMG) urethroplasty has also gained a success rate of 80%. Redo BMG urethroplasty has proven to be safe and feasible with good intermediate-term outcomes for recurrent anterior urethral strictures after previously failed BMG urethroplasty [5,12]. An elevated initial success rate of these interventions gives a higher probability of initially utilizing these curative procedures to avoid recurrences later.

The literature review has also regarded urethroplasty as the only curative option for patients in whom urethral dilations have failed [2]. This modality, in turn, is further considered as highly cost-effective after initial internal urethrotomy for recurrence. Long-term success rates are as high as 85%-90% for surgical reconstruction with urethroplasty. Studies further highlight the cost-effective analysis supporting the early use of urethroplasty (after one failed internal urethrotomy), given that the failure rate of urethrotomy increases substantially with repeated procedures [1,13]. 

## Conclusions

Therapeutic management of recurrent urethral strictures continues to appear like a challenging task. Despite a higher success rate of urethroplasty and temporary stent placement, urethral stricture recurrences are still an occurring entity. No definite therapeutic strategy has been adopted to evaluate and approach the morbidity effectively. Implementation of an effective primary procedure with minimally based complications should be generated to avoid future stricture recurrences. Studies on a larger scale need to be carried out on urethral stricture patients to obtain sufficient data to obtain a complete curative treatment option for the future eventually.
